# A Multimodal Deep Log-Based User Experience (UX) Platform for UX Evaluation

**DOI:** 10.3390/s18051622

**Published:** 2018-05-18

**Authors:** Jamil Hussain, Wajahat Ali Khan, Taeho Hur, Hafiz Syed Muhammad Bilal, Jaehun Bang, Anees Ul Hassan, Muhammad Afzal, Sungyoung Lee

**Affiliations:** 1 Ubiquitous Computing Lab, Department of Computer Science and Engineering, Kyung Hee University, Giheung-gu, Yongin-si, Gyeonggi-do, Seoul 446-701, Korea; jamil@oslab.khu.ac.kr (J.H.); wajahat.alikhan@oslab.khu.ac.kr (W.A.K.); hth@oslab.khu.ac.kr (T.H.); bilalrizvi@oslab.khu.ac.kr (H.S.M.B.); jhb@oslab.khu.ac.kr (J.B.); anees@oslab.khu.ac.kr (A.U.H.); 2 Department of Software, College of Electronics and Information Engineering, Sejong University, Seoul 05006, Korea; mafzal@sejong.ac.kr

**Keywords:** user experience evaluation, user experience measurement, eye-tracking, facial expression, galvanic skin response, EEG, interaction tracker, self-reporting, user experience platform, mix-method approach

## Abstract

The user experience (UX) is an emerging field in user research and design, and the development of UX evaluation methods presents a challenge for both researchers and practitioners. Different UX evaluation methods have been developed to extract accurate UX data. Among UX evaluation methods, the mixed-method approach of triangulation has gained importance. It provides more accurate and precise information about the user while interacting with the product. However, this approach requires skilled UX researchers and developers to integrate multiple devices, synchronize them, analyze the data, and ultimately produce an informed decision. In this paper, a method and system for measuring the overall UX over time using a triangulation method are proposed. The proposed platform incorporates observational and physiological measurements in addition to traditional ones. The platform reduces the subjective bias and validates the user’s perceptions, which are measured by different sensors through objectification of the subjective nature of the user in the UX assessment. The platform additionally offers plug-and-play support for different devices and powerful analytics for obtaining insight on the UX in terms of multiple participants.

## 1. Introduction

The user experience (UX) is a multi-faceted research area that includes diverse aspects of the experiential and affective use of a product, system, or service [1,2]. A UX assessment helps uncover the important aspects of designing high-quality interactive products and providing an overall positive UX [3]. The UX involves the user beliefs, preferences, thoughts, feelings, and behaviors when interacting with the product, system, or service [1]. It is thus subjective by nature, highly dependent on the use context [4], and linked to the potential benefit obtained from the product, system, or service [5]. The UX is measured using different constructs related to usability (perspicuity, efficiency, etc.), user perception (stimulation, dependability, novelty, etc.), and human emotional reaction [6] using various methods [7]. For example, a user’s feelings can be captured if the user “thinks aloud” while performing tasks. Similarly, the UX can also be interpreted by means of a daily diary over a certain period, such as a long-term diary study [8], day reconstruction method [9], repertory grid technique (RGT) [10], and experience sampling method (ESM) [11]. Additionally, the user can be observed by various means, such as a camera, sensor, user interaction tracker, and screen capture devices [7].

The subjective aspect of the UX, however, can make UX assessment difficult. Traditional methods of UX assessment rely on self-reported measurements, usability studies (performance), and observations [6,12], which may be unable to uncover the true user emotional experience [3]. A common method of expressing emotional and cognitive aspects is via retrospective self-reported verbal or written questionnaires [13,14,15], whereby the user is asked questions relating to their experience. However, this conventional method is highly subjective in nature and thus dependent on user interpretation, recollection, and bias [3]. Even when the questionnaire items are clear, most participants have difficulty engaging in honest and accurate introspection [3]; hence, they do not faithfully articulate their true emotions, abilities, and experiences. Meanwhile, open-ended interview methods [12] may avoid the confusion engendered by the specific-questioning process and can thus enhance the quality of user responses. Nevertheless, this method cannot completely solve the issues relating to self-reporting and self-discourse [16].

Observational methods can resolve the latter two problems, primarily when the user is unaware of the observation [17,18]. The user observation can reveal information relating to the task/user performance, efficiency, and errors while the user interacts with the system. However, both self-reporting and observation methods are unable to determine the psychological states of the user while employing the system. Both of these methods require skilled researchers for data recording, analysis, and interpretation, the latter of which is another source of subjectivity.

In short, determining how the user feels while employing the product, system, or service presents a significant hurdle for the UX evaluation. In reality, the user may have difficulty identifying, interpreting, and reporting their feelings and thoughts during or after use of the product, system, or service. Hence, in addition to self-reporting and observational measurements, UX researchers currently use physiological measurements to assess the user experience based on quantitative metrics.

In general, biometric sensors can detect emotional arousal and stress, motivation, and visual attention, states that have a direct relationship with user cognitive and affective conditions [3]. For instance, an eye tracker can detect visual attention [19,20,21], electroencephalography (EEG) can detect user motivations and emotional responses [22,23,24], the galvanic skin response (GSR) can measure stress and arousal through skin conductivity [25,26], and electrocardiogram (ECG) and electromyogram (EMG) can measure stress levels and muscle-arousing activities [27,28]. However, existing research has focused on limited methods and techniques to uncover the true experience of a user employing a product.

To address the limitations of the above individualistic methods and approaches, we propose an innovative “lean UX platform”, which employs a mix-method approach by combining observational, self-reported, and physiological measurements. It can evaluate the overall user experience over time by acquiring and synchronizing multimodal data while the user interacts with a product, system, or service.

The rest of this paper is structured as follows: in Section 2, UX evaluation methods of related work are described. In Section 3, the proposed lean UX platform architecture is generally described. In Section 4, the overall proposed platform is presented with respect to its architecture and implementation. In Section 5, execution scenarios are presented as case studies of “mind-mining” evaluations. Section 6 presents the evaluation and discussion, and Section 7 concludes the work.

## 2. Related Work

Many approaches have been proposed to acquire the user experience in various ways, including the questionnaire, facial analysis, vocal analysis, biometrics, and others. We classify these user experience evaluation methods (UXEMs) into three categories: (i) self-reported measurement, whereby the participant reports their feelings and thoughts in the form of a questionnaire, survey, or poll without expert intervention; (ii) observational measurement, a non-intrusive means of observing the user while interacting with the product, system, or service; and (iii) physiological measurement, whereby sensors are mounted on the user’s body for collecting physical information as quantifiable data. The following subsections detail the above categories.

### 2.1. Self-Reported Measurement

The self-reported approach has been used for a long time as a UXEM. Different tools have been developed to gather the self-reported data from users who express their feelings about the given product, system, or service [12]. No comprehensive solutions exist for extracting the holistic UX, and every method has its positive and negative aspects [5].

For emotion measurement via self-reporting in response to a stimulus, numerous methods have been used, such as the two-dimensional (2D) emotion space (ES) [13,29], to gather data by moving a mouse in the 2D space in response to valence and arousal. However, it cannot be applied to low-fidelity prototypes. Similarly, expressing experiences and emotions (“3E”) [14] uses a semi-structured method by providing a predefined template in which the user experience and sentiment data are entered as a daily diary. In addition, the day reconstruction method [9] is a well-known approach for capturing the user’s daily experience through their reporting of three important experiences or encounters each day. However, these methods are laborious and require researchers to analyze the gathered data [3,5,7,12].

Furthermore, the affect grid [15] provides a simple and easy scale for measuring affects in a 2D form, while the differential emotions scale (DES) [30] provides diverse categories of emotion to evaluate the user emotions. In addition, the Geneva emotion wheel [31] provides a wheel-shaped emotion scale through which a participant expresses their emotions, and PrEmo [32] uses cartoon animation to obtain the user’s emotional responses in the form of dynamic facial, body, and vocal expressions. However, the scale is subjective. The EMO2 [33] tool provides a rating scale in one and two dimensions for emotion measurement while using the product. Emocards and Emofaces [34] use a non-verbal, quick, and easy method that employs emotion cards (cartoon faces) indicating the user emotions while using the product. However, these approaches are intrusive during the given task.

Different questionnaires have been referenced in the literature for measuring various UX aspects, such as affect, aesthetics, attractiveness, pragmatics, hedonics, mental efforts, and satisfaction levels [6,27,35,36,37,38]. Lavie and Tractinsky [39] developed an aesthetics scale for website perceived aesthetics in terms of classic and expressive aesthetics. AttrackDiff [35] and User Experience Questionnaire (UEQ) [6] facilitate a rapid assessment of the user experience by obtaining the user’s expressed feelings, impressions, and attitudes after using the respective product. However, these assessments only indirectly reflect the experience, and do not focus on the actual experience. The mental effort scale [40] is an easy means of assessing how much effort is needed to complete a task; nevertheless, it requires other tools to obtain the holistic perspective. 

### 2.2. Observational Measurement

Observational measurement is an alternative approach to self-reporting or other methods of measuring user behavior. Situations exist in which the observational measurement method may be more scientifically valid than other methods when the participant is nonverbal or limited in his/her verbal or cognitive ability and is thus unaware and unable to report the behavior. Observational measurement enables detailed descriptions of behavior and its social and non-social contexts. Different methods and techniques, such as video-based facial expression analysis (FEA) [41], emotion from human voice [42], and tracking user interaction [43,44] by logging user actions have been employed for user experience assessment. 

Humans communicate considerable emotional information, both voluntarily and involuntary, through the movement of facial muscles. Facial expressions can be used in methods to understand a person’s emotional response and valence. Facial expression analysis detects muscle groups in action during different emotional responses, such as smiling, crying, and moving the inner and outer brows. Facial response provides a passive means of measuring a person’s experience. For example, the Facereader [45,46] software analyzes real-time videos for facial expression analysis by tracking the user emotional state during interactions with products or software. It also calculates the gaze direction, head orientation, and person characteristics. However, generate Facereader data are limited to six basic emotions: joy, anger, sadness, surprise, fear, and disgust. The relationship between the learning performance and user emotions expressed through the face was examined by Whitehill et al. [47], who found that a user smiles less when they learn more. Their findings show that a user smiles more when they feel embarrassed. In sum, FEA provides a useful approach to assessing affective responses of emotion valences. However, it is unable to identify emotional arousal.

Emotions can be recognized in the human voice using different statistical methods and voice features [42,48]. For example, anger can be detected from a high-pitched voice and faster speech rate. Numerous previous studies mentioned the most significant features for audio-based emotion recognition, such as intensity, duration, pitch, and spectral energy distribution [48].

Furthermore, analytical trackers are software systems that determine how the user interacts with the system. Several tracker systems can trace common user interactions, such as page tracking, event tracking, app/screen tracking, user time, exception tracking, custom dimensions, and metrics. For example, Google Analytics [43,49], Piwik [50], Appsee, and UXCam [44,51] are systems that can track common user interactions to assess the performance of a product by focusing on key performance indicators (KPIs) [52], such as daily active users (DAU), monthly active users (MAU), page views (PV), and unique visitors (UV). It can decipher the user context to better evaluate the performance of the use context, system, or product. However, these KPIs do not reflect the reason and emotion behind the user behavior.

Nevertheless, observational measurements have several challenges relating to the experience over time, and the measurement may vary on account of systematic or stochastic methods [53]. Additionally, observational methods are unable to determine the user’s psychological state while employing the system.

### 2.3. Physiological Measurement

In this section, we explore different biometric sensors, which obtain physical information as quantifiable data for the UX assessment. These tools can be used to validate the traditional measures or add extra information to the conventionally obtained data to extract the actual user perception of the product, system, or service. Herein, we briefly explore this specific research and related technologies.

Eye tracking is a powerful technology that tracks light corneal reflection and pupil dilation [54] for the identification of eye and gaze moments [19,20,21]. These data can be used to provide important insight that is unachievable from other techniques, such as user visual attention (locating a user’s eye positions) and distraction [19,20,21,55]. Eye tracking data reveal important information relating to arousal, engagement, fatigue, and interest because the eye is unable to deceive [3]. Thus, the issues relating to traditional measurement are avoided. User perception relating to tasks was investigated by Tzafilkou et al. [56] by using eye tracking data, such as eye fixation. These data were linked with user events while the user interacted with an interface; fixation duration, which was associated with user attention; and pupil size. Those authors used the gaze data to assess the self-efficacy and ease of use along with the questionnaire data. For example, a person gazing at the same point in the user interface felt more comfortable.

Similarly, Zheng [22] used eye tracking data with EEG signals to extract user emotions by fusing both the feature and decision levels to improve the emotion recognition model accuracy. The author used the pupil diameter as a metric for emotion classification, such as the pupil diameter changing in accordance with different emotional states. Stable patterns were also extracted for emotion recognition over time in both EEG and eye-tracking data. Meanwhile, Sanfilipp [57] used eye tracking data for tracking the user eye position and eye movements, while employing other biometric sensors for personnel training in situational awareness.

In addition to validating traditional methods, eye-tracking data provides information, such as how motion and background complexity can influence a player’s performance in a game environment. Eye fixation data are obtained while the user shoots in a game and the background complexity is measured. Moreover, eye tracking data can eliminate the obstacles of language or culture in UX assessments. For example, Sivajii [58] combined “think-aloud” data with eye tracking data (from multilingual country users) for website usability testing. Their findings showed that the results differed across different cultures on account of the “high-power distance”, that is, the unequal power distribution. In high-power distance cultures, feelings and thoughts are more likely to be less expressed, whereas low-power distance cultures are more open and more readily reveal their feelings and thoughts. Thus, eye tracking data remove these obstacles in true UX extraction while interacting with a website. In short, eye tracking technology assists the traditional UX assessment methods by adding validating and complementary data in the form of visual attention. 

Similarly, facial EMG [59] is used for the measurement of emotional states (e.g., arousal and valence) during gaming for positive improvement. However, facial EMG requires a proper laboratory setting and technical knowledge for handling artifacts, while engendering obtrusiveness and intimacy issues. Facial coding is another observational method for capturing behavior from facial expressions.

In the UX domain, multiple biometric sensors are used to detect affective information that can validate and complement the traditional methods. Each biometric sensor can detect a portion of the person’s behaviors. For example, eye tracking can detect visual attention. However, it does not provide adequate information on the user’s emotional states. Similarly, EEG and GSR [26,60] are effective at extracting the user emotional state in terms of arousal; however, they do not provide particular data relating to the emotional valence. GSR is a less effective method for measuring emotions. 

Thus, we conclude that one method’s weakness is the strength of another method. Consequently, for effective UX measurement, a mix-method approach is the best solution for extracting the true emotional experience. The mix-method approach provides more accurate and precise information about the user while the user interacts with the product, system, or service for the UX assessment. Nevertheless, this approach requires skilled UX researchers and developers to integrate multiple devices, synchronize data, analyze them, and produce informed decisions relating to the UX. Therefore, a single platform is required that can provide an integrated environment in a seamless manner with real-time synchronization and powerful visualizations for measuring the UX of any product, system, or service.

## 3. Lean UX Platform Architecture

**Figure 1:**
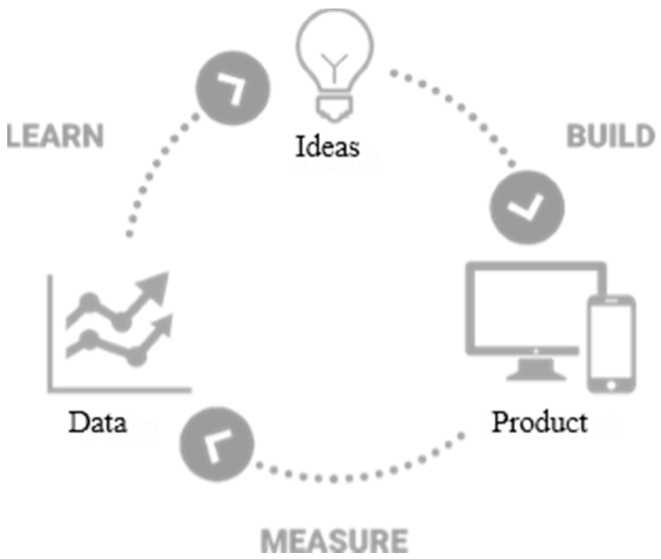
Lean UX learning loop.

Our proposed platform is based on the “lean UX” [61], which incorporates product development through the continuous measurement of a so-called “learning loop” (build—measure—learn), as shown in Figure 1. The main focus of the proposed platform is measuring and learning (inferencing), both implicitly and explicitly, from the subject’s usage behaviors and emotional responses. Consequently, UX research can be simplified by incorporating the human behavior research [5,62].

**Figure 2:**
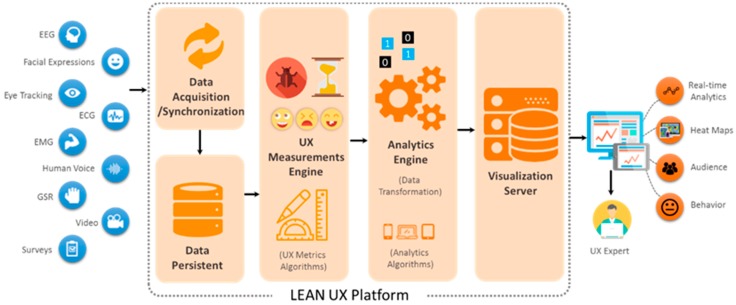
Proposed platform overview.

The proposed platform collects the user data through different methods and sensors, such as audio, video, and biometrics, as well as user interaction data and surveys, such as self-reported data use for the UX evaluation [7]. The abstract view of the proposed platform is shown in Figure 2.

**Figure 3:**
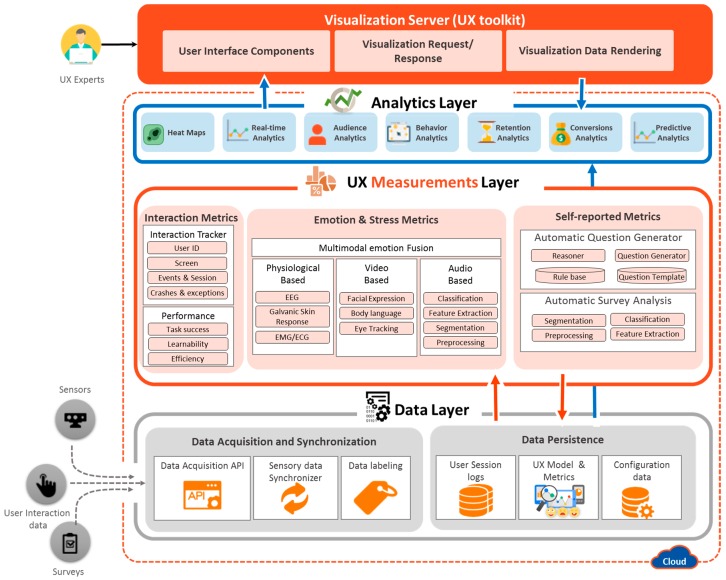
Lean UX platform architecture.

The platform is composed of four layers: the data layer (DL), UX measurement layer (UXML), analytics layer (AL), and visualization server (VS). The detailed architecture of the proposed platform is shown in Figure 3.

In short, the DL acquires and stores the data acquired from the multiple data sources, including audio devices, video equipment, biometric devices, surveys, and user interaction logs. The data acquired by DL is mainly employed by UXML to deduce the user’s emotion, perception, and usage experience. UXML deals with UX metric extractions of a particular phenomenon or object that will help quantify the UX of a person with respect to the product. The extracted information is then used by the AL upon the UX expert request to enable different types of analytics to infer the informed decision. The final layer is the visualization server, which serves as a toolkit for the UX expert to evaluate the digital product. It is a web-based application that is used to realize the different features, analytics, and visualizations based on UX measurement metrics of collected data.

## 4. Lean UX Platform Implementation Details

### 4.1. Data Layer

In the development of the “mining minds” platform, we created the data curation layer (DCL) [63,64] task to acquire, curate, and persist the data acquired from multimodal sources. We adopted the same DCL implementation for the Lean UX platform that collects multimodal data to determine the UX [65]. Data acquisition deals with the real-time data acquisition and synchronization obtained from heterogeneous data sources. A label is assigned according to the nature of the data and the data persists as a user session log for use by the UX measurement layer, which can then define the UX corresponding metrics. Upon receiving the UX metrics, they are calculated by the UXML and then stored in the data layer.

#### 4.1.1. Multimodal Data Acquisition and Synchronization

Data Acquisition and Synchronization (DAS) is a Representational State Transfer (REST; RESTful) web service that acquires real-time data from multiple data sources. After acquiring the data, synchronization is performed based on the time stamp of the device and queued based on the event for identification of the context. In the UX domain, every event is linked with a timestamp and heavily depends on the context [62]. All attached devices and sensors send data independently (they each have an independent clock); thus, a logical clock is needed to synchronize all attached devices to the Lean UX platform. Therefore, a synchronization mechanism based on a time frame—the so-called “complete and incomplete sync” [64]—is implemented. The details of the implemented algorithm are presented in [64]; it was developed using the Node.JS platform [64,66] to more efficiently handle events and non-blocking communication. After synchronization, each received data packet is labeled according to the data nature.

#### 4.1.2. Data Persistence

**Figure 4:**
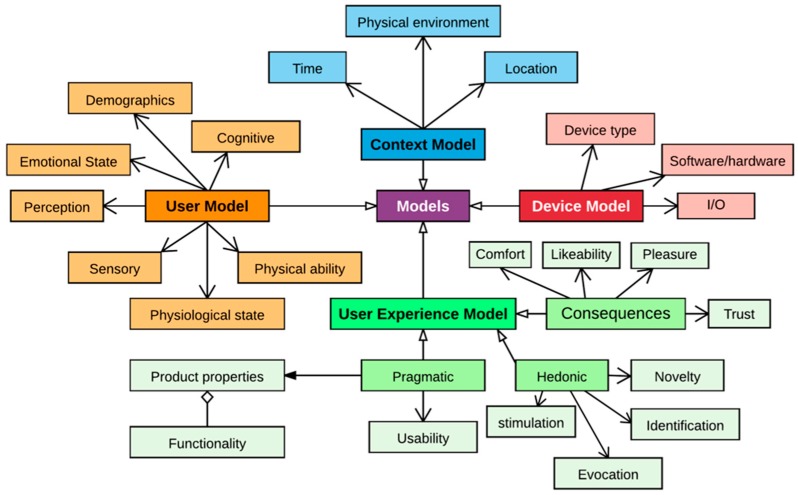
Lean UX Model.

Data persistence relates to two kinds of data persistence: relational database (RDBMS) and big data. RDBMS stores data related to the user model, which stores information related to user cognition, physical characteristics, sensory input, and UX. The context model stores information about the contextual factors; the device model stores information about different characteristics of the devices, such as screen resolution and their abilities of displaying content [67]; the UX model [68] stores the UX structural model [4]; and the configuration settings store information about the connected devices and experiment setting. An overview of the object model is shown in Figure 4. All the sensory data are directly collected from the devices and stored as big data for further analysis.

### 4.2. UX Measurements Layer

The UX measurements layer is the core of the lean UX platform for inference and modeling of the UX evaluation. It is composed of three main modules that deal with interaction metrics, emotion and stress metrics, and self-reported metrics.

#### 4.2.1. User Interaction Metrics

This module handles the collection of the user interactions and calculating the system performance. It monitors each user’s actions by determining how they use the application, problems they are experiencing, and how to resolve them. It views the application from the user perspective by pinpointing the performance, usability, and UX issues [69]. This module deals with real-time qualitative analytics along with traditional quantitative analytics (with numbers) by adding qualitative data on top of the quantitative data, thereby enabling UX experts to transform data into information, and information into insights.

The module consists of two main sub-modules: user behavior metrics and performance metrics. The user behavior metrics track common user interactions, such as page/screen, event, user timing, cross-domain tracking, tasks, crashes, exceptions, and custom dimensions [43,49]. The performance metric reveals how well users are using the product. It is also valuable in estimating the degree of a particular usability issue [70]. For example, if users are making several errors during a task, this means that there is room for enhancement. This module deals with the user interaction data collected by the analytics tracker during usage of the system, such as the task success, time spent on the task, errors, efficiency, and learnability.

#### 4.2.2. Emotion and Stress Metrics

**Figure 5:**
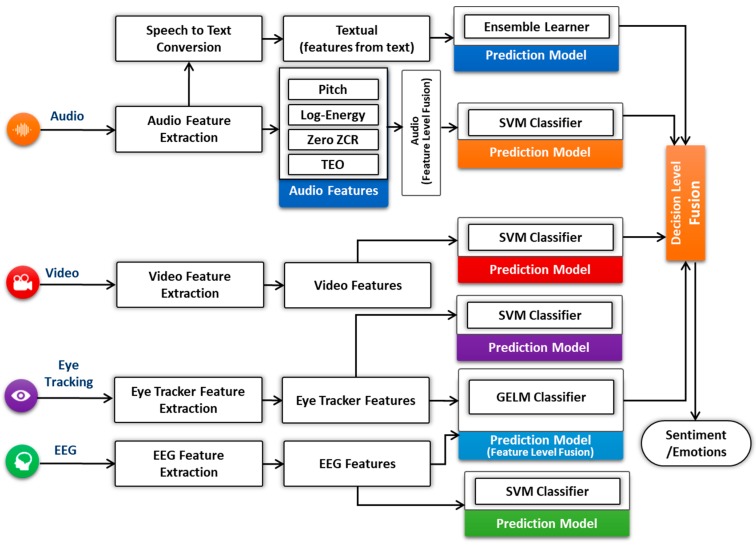
Hybrid level fusion for affect computing.

◯ *Physiological-based Emotion Recognition*: We use the biometric measurement to understand the emotional engagement of user while the user interacts with the system. We use multimodal data from various sensors, such as eye tracking, for visual attention and EEG for quick detection of emotions, motivations, engagement (arousal) in the cognitive workload and frustration level. We will add more modules that can measure emotional arousal and stress by the galvanic skin response (GSR) via measuring changes in the conductivity of the skin, and we will use EMG/ECG for the detection of muscle activity, motion, stress, and arousal. In this study, we implemented the eye tracking and EEG modules.
◯ *Video-based Emotion Recognition*: The video-based emotion recognition metric consists of two methods and sub-modules: facial expression analysis [41] and body language analysis. Automatic facial expression analysis (AFEA) plays an important role in producing deeper insights in human emotional reactions (valence), such as fear, happiness, sadness, surprise, anger, disgust, or neutrality. For AFEA, we used an inexpensive webcam to capture video of a participant in order to reduce the overall financial cost. Our developed AFEA first detects the face in a given video frame or image by applying the Viola Jones cascaded classifier algorithm. Second, different facial landmarks features are detected (e.g., eyes, brows, mouth, nose) as the face model. Finally, the face model is fed into the classifier to provide emotions and facial expression metrics as labels [41]. Non-verbal gestures (i.e., body language) play a significant part in the communication process and can yield critical insight into one’s experience while interacting with any computing system. We will use a depth camera to recognize emotions through user body language in upcoming version of lean UX platform release.
◯ *Audio-based Emotion Recognition*: We used an automatic method of measuring human emotions by analyzing the human voice collected through a microphone while using the system [71], such as anger, sadness, and happiness. The trained model is built on the emotion audio data collected from lab students using a microphone recording by manually labeling each audio clip, Berlin Emotional Speech (EMO-DB) [72], and SEMAINE corpus [73]. The model classifies incoming audio to the platform as seven basic emotions: fear, happiness, sadness, surprise, anger, disgust, or neutrality. A Voice Activity Detection (VAD) VAD technique that consists of short-time energy (STE) and short-time zero-crossing rate (STZCR) [74,75] is used to remove the background noise and eliminate the silent parts from audio signals. The speech signals are divided into frames, then STE detects the energy within each frame for voice segmentation. Afterward, STZCR is calculated from the rate of change of speech signal within a particular time window. These two features are used to extract the speech segment for emotion recognition and removed the unwanted frames from signals. The output of VAD is used by the audio feature extraction to extract the audio features such as pitch, log-energy, teager energy operator (TEO), and zero ZCR. Subsequently, we have employed the feature level fusion using a set of rules to choose the right emotions as a previous study [75].
◯ *Multimodal Data Fusion*: The primary goal of multimodal fusion is to enhance the accuracy of prediction classifiers [76]. It shows the importance of making a multimodal fusion framework that could effectively extract emotions from different modalities in human-centric environment. The benefit of using multimodal data from different devices is to get deep insights of human emotions and motivations. The platform fuses the different emotional measurements, such as audio, video, physiological, and eye tracking to acquire the complete overview of the user’s emotional experience by using the mixed method approach to measure the actual user’s emotional experience, as shown in Figure 5.

There are three different types of fusion level [76,77,78]: feature level, decision level, and hybrid level. Feature-level [79,80,81,82,83,84] is also known as early fusion, that fuses the features extracted from different modalities (e.g., audio, textual, EEG, and eye tracking) for prediction. Decision-level fusion [79,85] is called as late fusion, where the individual’s modalities classifier examined the features, gives the results, and then fuses the results to give a final decision. In feature-level, we combined the features of EEG and eye tracking (pupil size) for user sentiment recognition either positive, negative, or neutral. The combined feature vector has been used for the prediction classification. In decision-level fusion, we have employed the feature vector from each input modality and fed into individual classifier as shown in Figure 5. We have adopted the 10-fold cross-validation to estimate the performance of each recognizer. We have used the mean values of all prediction confidences score for prediction fusion. In our case, the rule-based approach has calculated the final label of the prediction as shown in the formula given below:

$$K=\;\mathrm{argmax}\;w_{1}C_{i}^{a}+\;w_{2}C_{i}^{v}\;+\;w_{3}C_{i}^{p}+\;w_{4}C_{i}^{t}),\;i=1,2,3,\ldots ,\;C\;\;=\;\left\{\mathrm{fear},\;\mathrm{happiness},\;\mathrm{sadness},\;\mathrm{surprise},\;\mathrm{anger},\;\mathrm{disgust},\;\mathrm{neutrality}\right\}$$

where *w*_1_, *w*_2_, *w*_3_, and *w*_4_ represent the weights of each prediction classifier. We have assigned the equal weights (0.1) to each classifier. *C* represents the classifier classes such as fear, happiness, sadness, surprise, anger, disgust, and neutrality, and $C_{i}^{a}$, $C_{i}^{v},$ $C_{i}^{p}$, and $C_{i}^{t}$ represent the confidences score for audio, video, physiological, and textual modalities respectively. The textual modality has used the same prediction model discussed in Section 4.2.3, after speech to text conversion. 

#### 4.2.3. Self-Reported Metrics

Self-reported metrics [35] deal with post-tasks that explicitly ask questions about the participant for information about their opinion and their interaction with the system, for example, overall interaction, ease of use, satisfaction, effectiveness, and efficacy. It consists of two main modules: automatic question generation and automatic survey analysis. Automatic question generation asks questions based on UX measurement information that triangulates [86] stated answers with biometric unconscious responses. The reasoner [87] component uses the UX measurement information as input data, which are quantified by emotion and stress metrics and interaction metric modules. Based on input facts, the reasoner fires the rules. The fired rules are passed to the question generator, which uses the predefined question templates to ask selective questions against the post-task performed by the participant.

The rule base was constructed from the existing standardized usability and UX questionnaires, including AttrakDiff [35], User Experience Questionnaire (UEQ) [6], Questionnaire for User Interaction Satisfaction (QUIS) [36], Single Ease Question [88], Software Usability Measurement Inventory (SUMI) [37], and Software Usability Scale (SUS) [38]. The production rules “IF-THEN” was used to associate the selected questionnaires with post-task UX measurements from user observational data. First, we extracted all questions of bipolar words and merged the duplicate one, arranged it as an LTR (negative to positive), and assigned an ID to each bipolar word that uses an index, as shown in Appendix Table A1, to load the bipolar word based on the reasoner action. Accordingly, the question template is filled by the question generator module. The partial list of candidate rules is presented in Table 1.

We created predefined templates that store the question template repertory by ID, such as T1. One sample question template structure that uses the question generator component is the following:

**I was** ___________________ **with the** ______________ **complete the task.**

The question generator selects and completes the template based on the resultant fired rules, e.g., R1, R3, and R4 based on the UX measurements facts.

**Example** **1.** I was feeling annoyed with the confusing UI to complete the task.

**Example** **2.** I was feeling unfriendly with the unpleasant UI to complete the task.

**Example** **3.** I was pleased with the time taken to complete the task.

**Figure 6:**
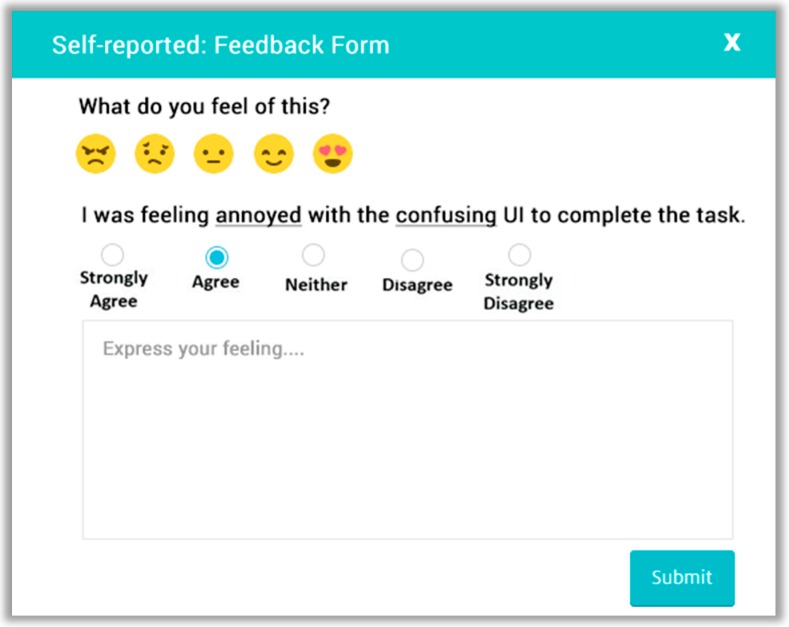
Self-reported feedback form.

Additionally, the question generator adds a free text field, user emotions Likert scale emoticons as shown in Figure 6 and then sends it to the participants for obtaining the response. The obtained user’s response is persisted in the database for analysis.

The automatic survey analysis deals with the analysis of closed-ended and open-ended questionnaires. Analysis of the former deals with the response transformation, measurement of central tendency, variance, confidence interval, and scale consistency by assigning the questions items to UX model. For example, word annoying belongs to the “attractiveness”, and “confusing” belongs to the “perspicuity” of UX scale. Based on that UX scale, UX moderator evaluates the UX of the project.

The latter analysis deals with the free text user responses. First, it loads all user free text responses, which are preprocessed before applying the topic modeling using Latent Dirichlet Allocation (LDA). LDA is an unsupervised generative statistical model, which assumes that each document may be consisted of different topics and words distribution over each topic. We implemented the pyLDAvis (https://github.com/bmabey/pyLDAvis), a python library for interactive topic model visualization for the extraction of topics from the collected user’s feedback. All the collected user’s textual feedbacks are processed by preprocessor module to remove the numeric data, erase punctuation, remove stop words, convert text into lower case, and stemming. We set number of topics to 30, 50 number of words per topic, and 1000 number of iterations to interpret the results. LDA extracts the topics and assigns a topic name based on dictionary words. Based on the collected topics on a different project, we will extract the important UX constructs/dimensions, for the inclusion in the UX model.

**Figure 7:**
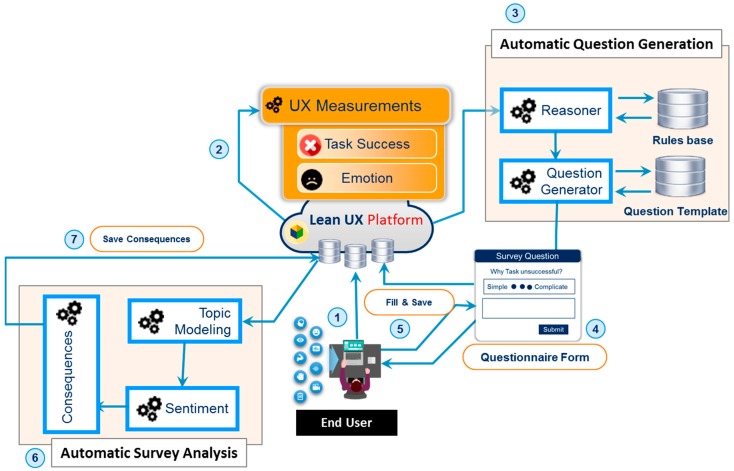
Survey workflow: triangulation of UX metric with self-reporting.

We have built the classifiers to classify the user textual feedback either as positive or negative along with emotions using automatic survey analysis module. Both positives and negatives user feelings are related to the post-task for determining the UX consequences. The overall workflow of the aforementioned process of the self-reporting metric is shown in Figure 7.

**Figure 8:**
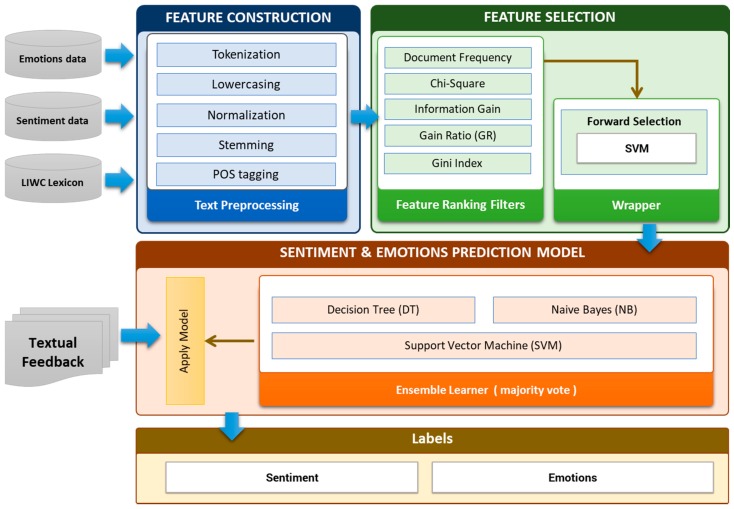
The workflow of sentiment and emotion analyzer.

The workflow of sentiment and emotion analyzer is shown in Figure 8. The workflow consists of three main steps (a) Feature construction (b) Feature Extraction and Selection (c) Learning of prediction model. The details of these steps are described in the subsequent sections.

(a) Feature construction

In text classification, conversion of text into feature vector is an essential task. The construction of an adequate feature space from the raw and unstructured text for better learning performance is necessary for text classification. It is essential to include only relevant/appropriate features for text representation. In the recent literature, different features representation methods have been used to represent text, for textual classification. These are bag-of-words (BOW), linguistic patterns using part-of-speech (POS) tags, high order n-gram features (character n-grams and word n-grams), dependency parsing tree, semantic features (lexicons and dictionaries) and structural features [89,90]. In this study, we used BOW, POS tags, semantic features (lexicons and dictionaries). For feature construction, we have applied preprocessing step to make the initial feature vectors which are suitable for further feature extraction and selection process. The preprocessing step contains tokenization, stop-word removal, and stemming (Porter algorithm). We used PENN Treebank scheme [91] for POS tagging pattern. For example, the feature “excellent interface” filtered by the POS tag pattern “JJ NN” and “was disappointed” feature is filtered out by the pattern “VBD VBN”. TF-IDF term weight scheme have been applied for word vector creation.

(b) Feature Selection

**Figure 9:**
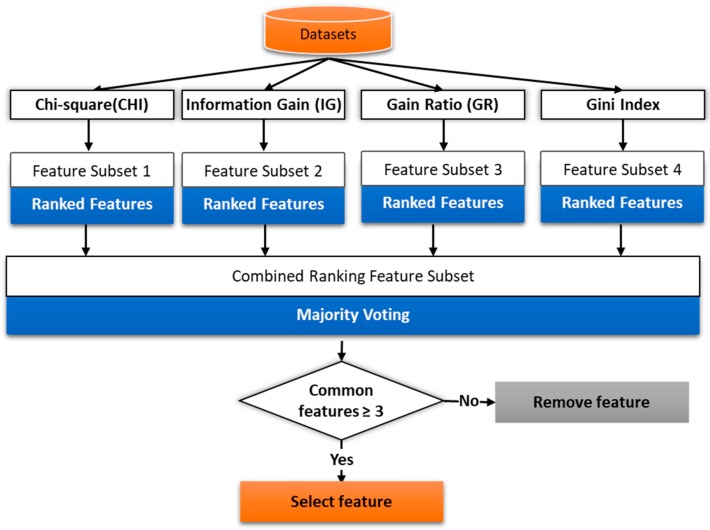
Filter base feature selection process.

Feature selection is the way to extract and select the most important and relevant features. It reduces the dimensionality feature space without losing too much information for an accurate prediction. The selected features are used to train the predictive model. We have employed filter method and wrapper method for effective features selection. In the filtering method, the subset of important features/relevant features is selected by ranking them according to specific scoring schemes based on the intrinsic properties of the features. The low scoring features are removed while highest scoring features are selected. The filter uses a fast evaluation function and is independent of the classifier. In the filter based method, we have used the filters like chi-square, Gini index, gain ratio, and information gain as shown in Figure 9.

The word-vector is input in feature selection module. The individual filter assigns weight to each feature using their internal logic and select the initial subset features. We apply the majority voting method for the final feature selection. We set the threshold value to 3 that checks for common features selected by at least three filters. Then we have applied the wrapper method (forward selection process), in subset feature selection. In wrapper method, various subsets of features are generated and evaluated. The forward selection starts with an empty selection of features/attributes and, in each iteration, it adds new attribute of the given recordset. We have applied 10-fold cross-validation using SVM learner to estimate the performance, if the added attribute gives the higher performance then is added to the selection. Then a new round is started with the modified selection. We have added the stopping behavior to stop the iteration if no significant increase in performance.

(c) Learning prediction model (Ensemble Learner)

We have employed the ensemble learning method for sentiment and emotion classification. Ensemble learning combines the predictions of multiple base learners to improve performance over a single learner. In this work, we have employed majority voting technique in conjunction with three base learners namely, Support Vector Machine (SVM), Naïve Bayes (NB) and Decision Tree. Based on the majority voting of base learners, the user textual feedback is classified into either positive or negative class along with basic emotions (Joy, anger, fear, sadness, and surprise).

### 4.3. Analytics Layer

The analytics layer is responsible for providing different analytics based on the UX expert query. It is composed of a heat map, real-time, audience, behavior, retention, conversion, and predictive analytics. Heat-map analytics provide a comprehensive solution to present user interaction data in a more intuitive way. We used the Heatmap.js library to create a heat map from the eye tracker fixation metric and analytical tracker using click data, which helped us determine the obstructions and ignored parts in the user interface.

The audience analytics module uses the subject’s data to understand the audience habits and determine what makes them more or less likely to take the action in a system. The behavioral analytics module deals with how and why the user acts based on the retrospective analysis. The retention analytics module checks how often the user returns to the product/application in a specific time frame and to check if either the user increases the frequency with changes in the user interface.

Conversion analytics module measures the user state of change in terms of the conversion rate, checks the cause of each process of success or failure, and determines why a user failed to complete certain tasks. Based on this information, we can identify the main hurdles that the user faces while completing some specific steps in the application and how to overcome those hurdles. Predictive analytics make predictions about user’s next move by using supervised machine learning to forecast the next move based on user observational data.

### 4.4. Visualization Server (UX Toolkit)

**Figure 10:**
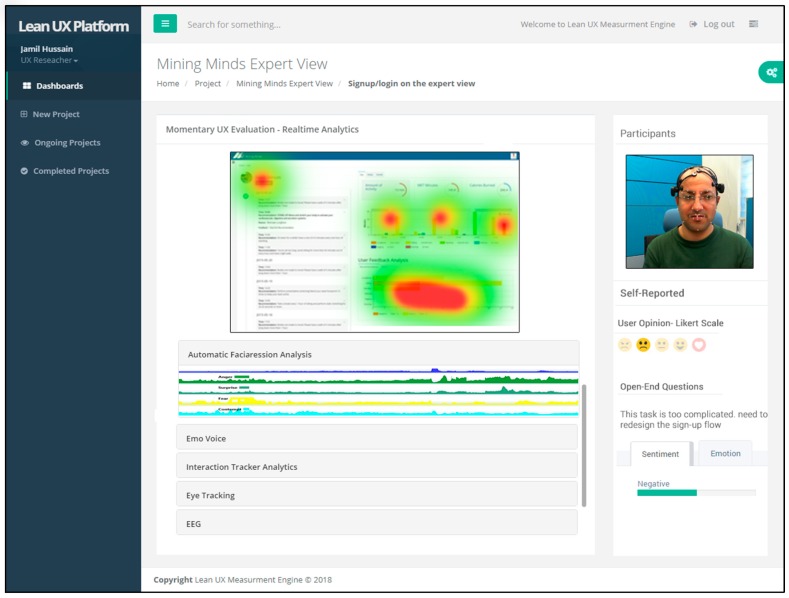
User Interface of UX toolkit.

The visualization server is a client application that is used by the UX expert to evaluate the product, system, or service. It is a web application for realizing the different features, analytics, and visualizations based on UX measurement metrics and collected data. The UX toolkit is designed as responsive and adaptive so that it can operate on any device and operating system. The toolkit user interface is shown in Figure 10. We developed the toolkit using the Django platform. For markup language, HTML 5 along with JavaScript libraries, such as D3.js, were used. For API design, the Django rest platform was used. The Lean UX toolkit evaluates the product with respect to momentary, episodic, and cumulative UX based on the study design. It provides plug and plays support to attach sensors and devices according to the design study. Before collecting the multimodal user interaction data, the application must be registered to the Lean UX platform through the UX toolkit, and SDK code should be added to the application with assigned registered code. From that point, the UX expert can check the real-time visualization that is generated by analytics based on collected data to evaluate the momentary UX. The UX expert can also evaluate the episodic and cumulative UX in a retrospective manner. It also provides access to all the question templates and rules to modify according to the application. The rest of Lean UX toolkit workflow and screenshots are presented in Appendix B.

## 5. Execution Scenarios as Case Studies of Mining Minds Evaluation

In this section, a conceptual case study is discussed. The Ubiquitous Computing Laboratory (UCLab) development team developed a platform related to health and wellness named “Mining Minds” [63]. The product is concerned with the well-being and interaction of the users. We evaluated the Mining Minds expert view through the LEAN UX platform.

**Figure 11:**
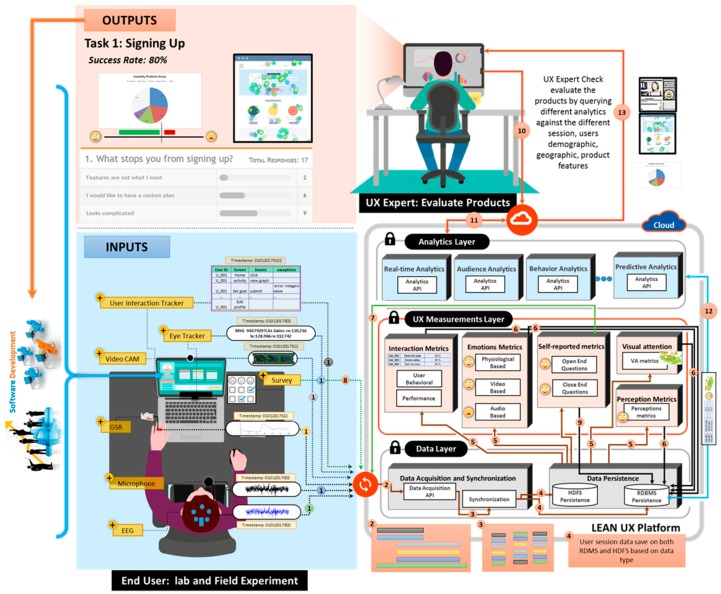
Overall workflow of the proposed platform.

First, we set up an experiment through the Lean UX toolkit. Multiple sensors were connected to obtain a response in the form of user interactions, video, audio, EEG, and eye tracking. These sensors sent data to the lean UX platform whenever participants used the Mining Minds application. The multimodal sensory data were gathered, synchronized, and labeled before persistence and routing to different UX measuring modules. Depending on the data size and nature, the data persisted either as big data or in a relational database. Each measuring module analyzed the user stimulus and measured the metrics of the UX. The self-reporting module automatically generated the UX questionnaire. The results of the measurement confirmed the user responses. The overall workflow of the proposed platform is shown in Figure 11.

**Figure 12:**
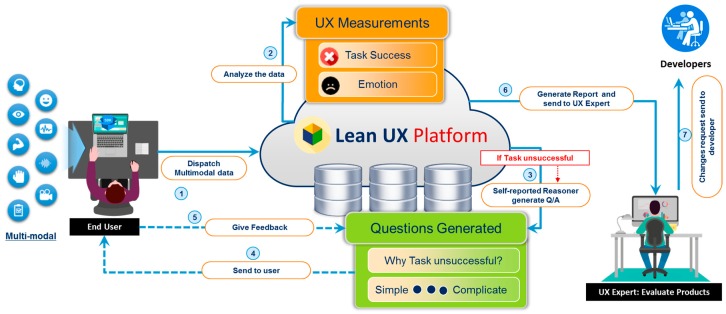
How it works.

After an expert-defined duration, we investigated the user experience in three modes: momentary, episodic, and cumulative, through the Lean UX toolkit. We evaluated different UX results based on queries. All concerning analytical modules of the analytics layer generated interactive representations of reports in the form of graphs based on UX measured metrics. Furthermore, UX experts can evaluate and decide the area of improvement of that product and then indicate it to the development team. An abstract view of the case study is shown in Figure 12.

## 6. Results and Evaluation

The proposed platform was evaluated from different aspects, such as multimodal data acquisition error rate, synchronization accuracy, individual UX measurements metrics ranging from interactions, multimodal emotions recognizers, and self-reported assessments.

*Sample*: The 10 participants were university students (70% male and 30% female), and their ages ranges from 19 to 44 (29 mean). They had a mixed race and were used for evaluation of the Lean UX platform. Each session was 20 min on average. The data were collected from different devices, e.g., EEG, camera, eye tracking, interaction tracker, text analyzer, and microphone. The results and discussion of evaluations are presented further below.

### 6.1. Multimodal Data Acquisition and Data Synchronization Process

The accuracy of the data acquisition and synchronization process was validated by connected different devices, such as EEG, camera, second-generation Kinect, eye-tracker, and PC with the Lean UX platform cloud. All data streams from multimodal data sources were acquired, synchronized at server endpoints, and checked for data accuracy using a three-second window size. The rate of missing data packets was used to measure the accuracy of data acquisition module shown in Table 2. The results show a 0.03% average error rate, which is very low, meaning that the platform acquired and processed multimodal data safely.

**Figure 13:**
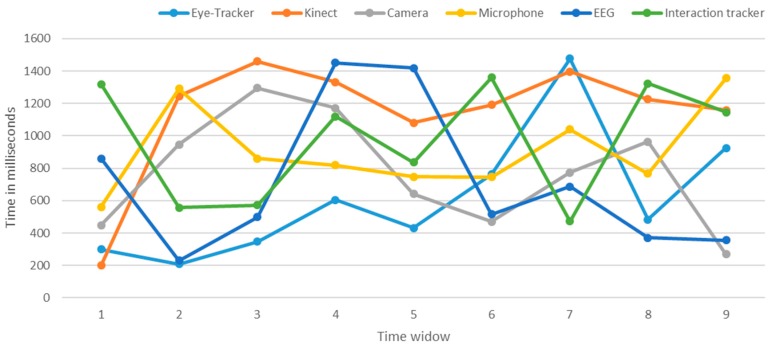
Multi-modal data sync testing per time-window.

The results are shown in Figure 13. The multimodal data from all devices were effectively synchronized at cloud endpoints in milliseconds. The synchronization module synchronized all sensors, stimuli and API data streams in real-time without manual post-synchronization of data. For example, the eye-tracker, Kinect, camera, microphone, EEG, and interaction tracker communicated at 300 ms, 200 ms, 450 ms, 562 ms, 860 ms, and 1318 ms, respectively, at the first window frame. The synchronization module recognized that all incoming data streams belonged to the single event. The results depicted that all data streams were well synchronized in a real-time manner, showing the perfection of the synchronization module. 

### 6.2. Emotion and Stress Metrics

**Figure 14:**
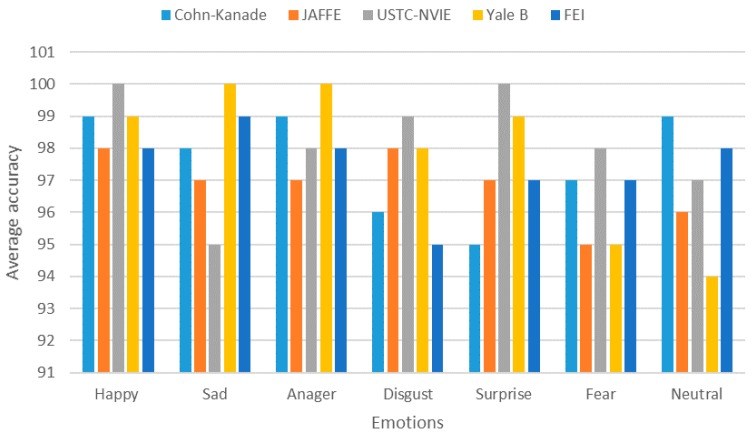
Recognition average accuracy for each dataset.

*Video-based emotion recognition*: We evaluated the video-based emotion recognition component by using five datasets: Cohn-Kanade dataset [92], JAFEE dataset [93], USTC-NVIE dataset [94], Yale B face dataset [95], and FEI face dataset [96]. Table 3 shows the confusion matrix of automatic facial expressions for Cohn-Kanade dataset. Figure 14 shows the average accuracy for each dataset. The results show a high accuracy for the happy, anger, sadness and surprise, while relatively low accuracy for the fear and disgust. Additionally, fear and disgust were mixed with sadness and anger owing to the subject’s expressions. However, generally, the model accuracy was quite reasonable compared to the other video-based emotion recognizers. There were some challenges for effective emotion recognition for heterogeneous populations with respect to demographic, cultural, and impairment aspects, which can be resolved by improving the landmarking techniques to classify the emotions for face impairment. 

*Audio-based emotion recognition*: The result of audio-based emotion metric extraction is shown in Table 4 for Emo-DB dataset. The results show a high accuracy for anger and surprise, while a relatively low accuracy is shown for happy and disgust. Additionally, happy and anger were mixed owing to the high sound pitch, while sadness and neutral were mixed owing to the soft voice. However, generally, the model accuracy was quite reasonable compared to the other audio-based emotion recognizers. There were some challenges, such as tone differences and voice pitch, which made the audio-based emotion recognition difficult.

**Figure 15:**
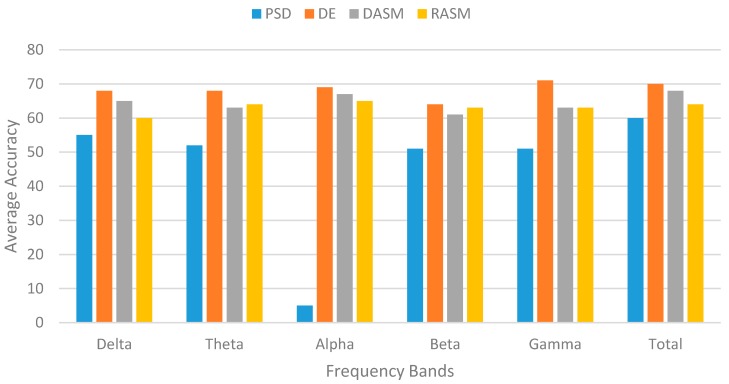
Average accuracy of the classifier using different features on different frequency bands.

*EEG-based emotion recognition*: For the EEG-base emotion recognition, we used four features—differential entropy (DE), power spectral density (PSD), rational asymmetry (RASM), and differential asymmetry (DASM)—to extract the most stable pattern for emotion recognition either positive, negative, or neutral. The results are shown in Figure 15, where DE achieves a higher accuracy for all frequency bands compared to the other features. From the experiment and results, we identified that the DE feature is more suitable to fuse with other features of emotion recognizers, such pupil size of the eye tracking data.

**Figure 16:**
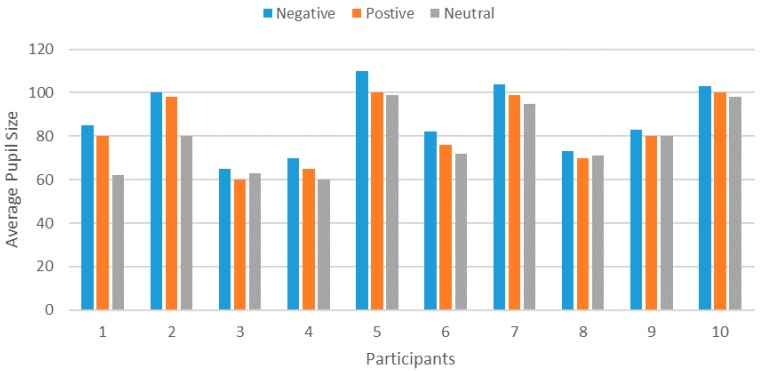
The average pupil size of each trail.

*Pupil Diameter*: We performed different experiments based on the pupil size metric using an eye tracker to observe how the pupil size changed in accordance with different emotional states. From the experiments, we found that the pupil size increased (dilated) in a sorrowful state, and was smallest in a calm state, as shown in Figure 16.

For both positive and negative emotions, the pupil size was larger compared with neutral, which showed a correlation with different emotions. We extracted different features: power spectral density (PSD) and differential entropy (DE) from the pupil size to measure the emotional arousal, and the DE feature outperformed PSD.

*Emotion fusion*: Table 5 shows the average accuracy of emotion fusion. The results show that fusion accuracy is higher than the individual classifier accuracy. Furthermore, we used the paired *t*-test (*p* < 0.05) to evaluate the accuracies of all the methods. The *t*-test analysis showed no significant differences between the feature level and decision level fusion.

### 6.3. Self-Reported Metric

The open-ended question analyzer module assessed the affective content (sentiment and emotions) by using lexicon-based dictionaries; POS-tagging; bag-of-words; and in combination with classifiers, such as SVM or NB. We used multiple lexicon dictionaries (e.g., LIWIC, and Custom), and annotated a training dataset at different levels—document, paragraph, sentence, and word level—to help extract the true emotions from the user textual response. For feature selection, filter and wrapper approaches were used for the selection of optimal features that improved the classification accuracy. For the experiment, we used five datasets that are widely used for text-based sentiment analysis. The results of experiments shown in Table 6 reveal that the ensemble method with minimal feature selection strategies can effectively increase the accuracy of classification compared with the baseline classifier. 

## 7. Conclusions

Understanding user feelings, thoughts, and needs are very important to engaging, sustaining, and increasing the purchase of a product, system, or service. The UX assessment reveals the user feeling about the product, system, or service and their functionalities. The user may have difficulty expressing their feelings and thoughts about a product, system, or service through traditional methods. Sometimes they may be unable to interpret their own feelings in order to describe them. The physiological measurements in assessing UX can detect emotional arousal and stress, motivation, and visual attention that have direct relationships with user cognitive and affective states in a non-intrusive way. The mixed-method approach showed importance in the UX evaluation methods by providing more accurate and precise information about the user while interacting with the product.

However, this approach requires a skilled researcher to integrate multiple devices, synchronize data, analyze data, and make informed decisions. Thus, we developed the Lean UX platform to provide an integrated environment in a seamless manner with real-time synchronization and powerful visualizations. The platform offers plug-and-play support for data collection from different devices and powerful real-time analytics visualization to enable insights of time spans of the user experience with multiple participants. Further, it helps identify the areas of improvement after assessment of any product, system, or service to improve the overall UX.

However, improvements can be made in terms of the classifier performance. We will add more classifiers and biometric sensors, such as GSR and ECG/EMG, to the Lean UX platform to extract the true user emotional experience. Finally, we will increase the datasets for effective emotional state recognition. 

## Figures and Tables

**Table 1:** A partial list of candidate rules.

Rule ID	Condition (IF)	Action (THEN)
R1	**IF** emotional_state = “anger” **AND** congnitive_state = ”stress” **AND** usability.tasksuccess = “failure”	T1, WL1, WL13
R2	**IF** emotional_state = “anger” **AND** congnitive_state = ”confuse” **AND** usability.tasksuccess = “failure”	T1, WL1, WL21
R3	**IF** emotional_state = “disgust” **AND** congnitive_state = ”confuse” **AND** usability.tasksuccess = “failure”	T1, WL19, WL21
⋮	⋮	⋮
Rn	**IF** emotional_state = “happy” **AND** usability.tasksuccess = “complate”	T1, WR14, WR9

**Table 2:** The data acquisition process accuracy.

No. of API Calls	Missing Data Packets	Error Rate
20,000	2	0.010
40,000	5	0.012
60,000	9	0.015
80,000	12	0.015
120,000	21	0.017
Average		0.03

**Table 3:** Facial Expression confusion matrix using Cohn-Kanade dataset (unit %).

Expression	Happy	Anger	Sad	Surprise	Fear	Disgust	Neutral
Happy	99	0	0	1	0	0	0
Anger	0	98	0	1	0	1	0
Sad	0	0	98	0	1	0	1
Surprise	0	1	1	96	0	2	0
Fear	0	1	1	1	95	1	1
Disgust	0	1	1	0	1	97	0
Neutral	0	0	1	0	0	0	99
**Overall Accuracy**	97.429%						

**Table 4:** Audio base emotion recognition confusion matrix using Emo-DB dataset (unit %).

Expression	Happy	Anger	Sad	Surprise	Fear	Disgust	Neutral
Happy	83	10	0	7	0	0	0
Anger	2	92	0	1	0	5	0
Sad	0	0	87	0	2	0	11
Surprise	6	3	0	89	0	2	0
Fear	0	1	1	8	87	1	2
Disgust	0	7	2	6	2	80	3
Neutral	0	0	10	0	2	0	88
**Overall Accuracy**	86.571%						

**Table 5:** The average accuracies of each classifier and fusion method.

Subject	Facial Expression	Audio Base	Textual	EEG (DE)	Eye Tracking	Fusion	
						Feature Level	Decision Level
1	95	84	91	68	80	96	96
2	92	82	89	63	82	97	98
3	100	80	94	64	83	98	99
4	98	83	89	62	89	93	98
5	98	84	93	76	90	92	93
6	90	83	94	70	81	97	98
7	94	84	94	72	87	91	93
8	93	83	91	69	85	94	94
9	93	80	92	64	80	95	93
10	98	82	92	70	87	98	96
**Average**	**95.1**	**82.5**	**91.9**	**67.8**	**84.4**	**95.1**	**95.8**

**Table 6:** Average accuracies of each classifier for each dataset.

Dataset	Classifier	# of Features	Accuracy
Movie	SVM	3625 ± 1209	93
	NB	2400 ± 1375	92
	DT	3816 ± 1254	88
	Ensemble	3779 ± 1314	**94**
	**Average**	**3405**	**91.75**
Book	SVM	2199 ± 1066	87
	NB	2612 ± 1074	86
	DT	2031 ± 1048	83
	Ensemble	2956 ± 1021	**89**
	**Average**	**2449**	**86.25**
Electronic	SVM	1323 ± 474	85
	NB	1002 ± 1090	**89**
	DT	1938 ± 625	87
	Ensemble	1760 ± 855	86
	**Average**	**1505**	**86.75**
Kitchen	SVM	1843 ± 770	89
	NB	1566 ± 470	86
	DT	1600 ± 787	89
	Ensemble	1969 ± 877	**90**
	**Average**	**1744**	**88.5**
Music	SVM	642 ± 296	**89**
	NB	819 ± 276	87
	DT	855 ± 267	86
	Ensemble	362 ± 155	88
	**Average**	**669**	**87.5**

**Figure A1:**
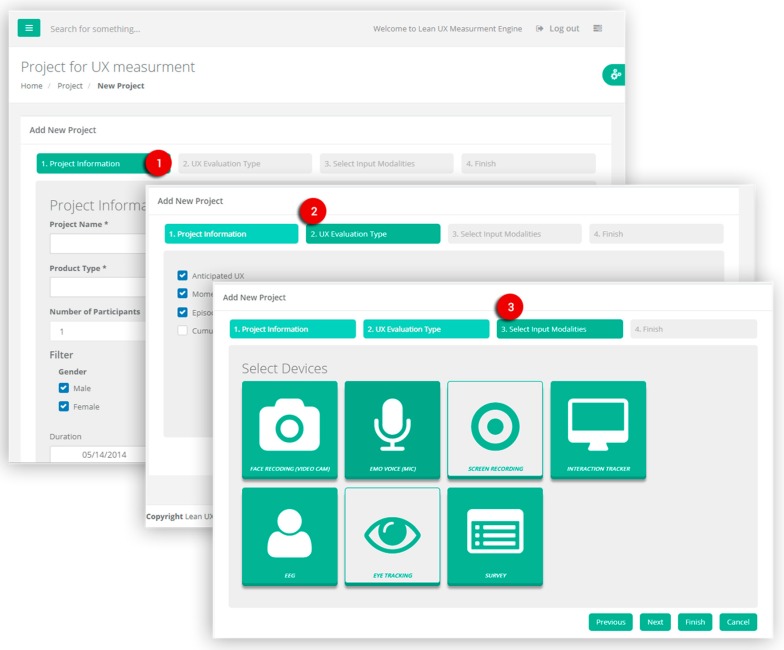
Project creation step-wise process.

**Figure A2:**
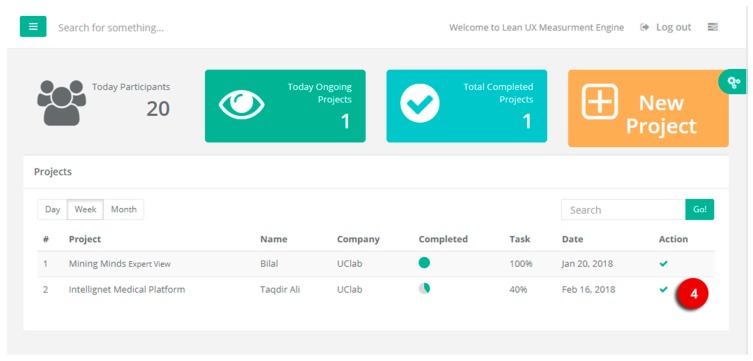
Dashboard of Lean UX- List of created projects for UX evaluation.

**Figure A3:**
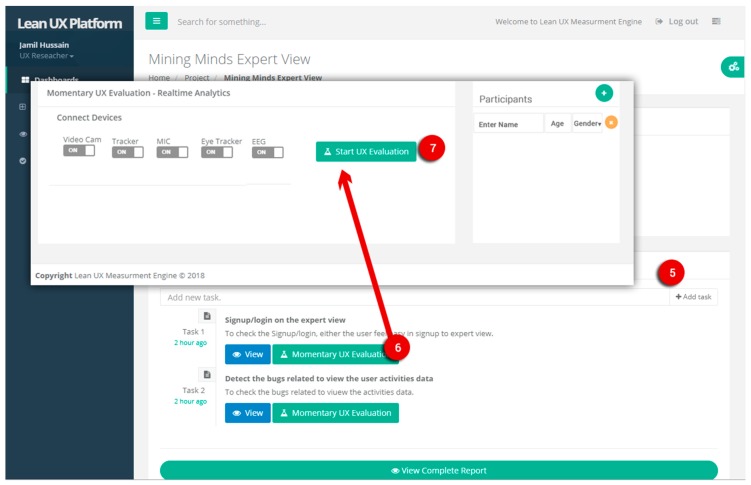
Task creation and evaluation process by task wise.

**Figure A4:**
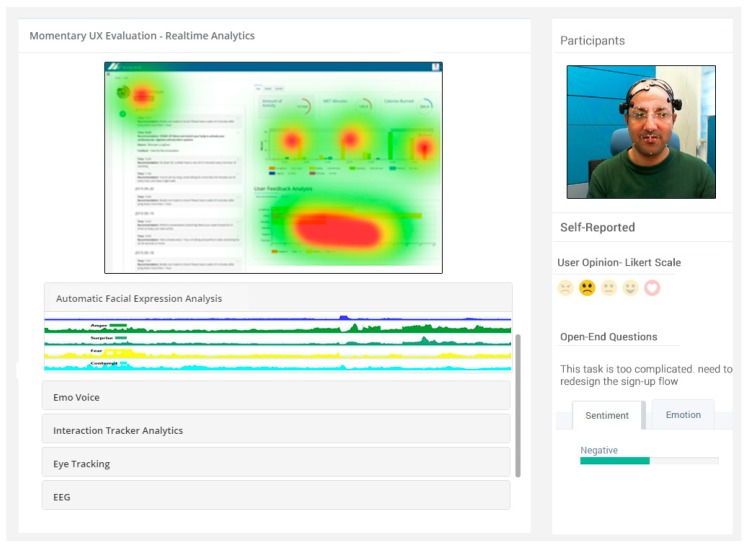
Momentary UX evaluation: real-time data collection and UX metric measurement.

**Figure A5:**
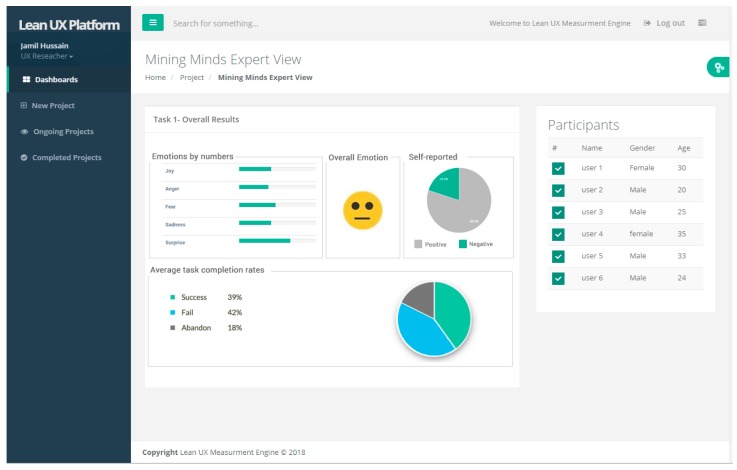
Task wise result view.

**Figure A6:**
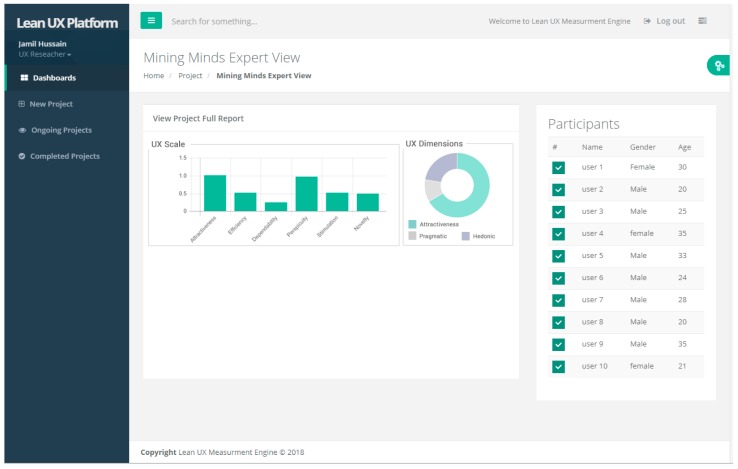
Project wise result view.

## Appendix A

Table A1 shows the selected items from existing UX questionnaires.

**Table A1:** The average accuracies of each classifier and fusion method.

Question ID	Bipolar Words	
	WL	WR
1	annoying	enjoyable
2	not understandable	understandable
3	dull	Creative
4	difficult to learn	easy to learn
5	inferior	valuable
6	boring	exciting
7	not interesting	interesting
8	unpredictable	predictable
9	slow	fast
10	inventive	conventional
11	obstructive	supportive
12	bad	good
13	complicated	easy
14	unlikable	pleasing
15	usual	leading edge
16	unpleasant	pleasant
17	not secure	secure
18	motivating	demotivating
19	Does not meets expectations	meet expectations
20	inefficient	effient
21	confusing	clear
22	impractical	practical
23	cluttered	organized
24	unattractive	attractive
25	unfriendly	friendly
26	conservative	innovative
27	technical	human
28	isolating	connective
29	unprofessional	professional
30	cheap	premium
31	alienating	integrating
32	separates me	brings me closer
33	unpresentable	presentable
34	cautious	bold
35	undemanding	challenging
36	ordinary	novel
37	rejecting	inviting
38	repelling	appealing
39	disagreeable	likeable

## Appendix B

Appendix B depicts the lean UX platform toolkit. The moderator first login into the Lean UX platform toolkit, to create a new project by clicking on “Add new project” button. The details of project should be entering in step-wise form such as project information, UX evaluation type (anticipated UX, momentary UX, episodic UX, and cumulative UX), and input modalities/stimuli (video cam, MIC, screen recording, interaction tracker, EEG, Eye tracking, and survey) shown in Figure A1. The input modalities are dependent on the UX evaluation type. For example, if moderator selects only anticipated UX, then the evaluation will be performed using “survey”. In the survey, we are using User Experience Questionnaire (UEQ) scale to collect the user experience for measuring the UX, contains six dimension scales such as novelty, stimulation, attractiveness, dependability, and efficiency. While for the other type of UX evaluation, all types of input modalities will be available. The moderator can select any type of input modalities, depend on their study.

After successful creation of the project, the moderator can add tasks to project as shown in Figure A2 and Figure A3. The system will generate automatically the project Id, which is used by the interaction tracker module, to track the user interaction as discussed in Section 4.2.1. The moderator first adds the JavaScript code in the header of each page by assigning the project id. The JavaScript code is also responsible to display the feedback form on the completion of the task or error situation.

The moderator can collect the UX measurement data by connected the sensors, sensors connectivity is auto checked by the system. The moderator should add the participant information by adding their name, age, and gender. By clicking on “Start UX evaluation” button, all measurement modules will start collecting the data and perform real-time UX measures related to emotions, user interaction, and self-reported as shown in Figure A4.

The moderator can check the different modalities measures such as automatic facial expression analysis, emo voice, interaction tracker analytics (e.g., the heatmap of user click and mouse move data), eye tracking, and EEG. At the successful/unsuccessful of task, the self-reported feedback form will be appeared on the participant screen to collect the self-reported feedback. The participants can express their feeling in both Likert scale and free text format. The self-reported data will be available on the submission of self-reported form by the participant to the moderator. The open end question analyzer will analyze the free text self-reported feedback to extract the user sentiment and emotions related to that task shown in Figure A4. This evaluation process will repeat for all participants who will participate in the study for each task. The moderator can check the results of UX evaluation at the task level and project level as shown in Figure A5 and Figure A6.

The moderator can check the results by applying the participant’s filter such as emotions by numbers (fusing the different modalities emotions), overall emotions, self-reported sentiment, and task completion rates.
